# The discovery of the BABA receptor: scientific implications and application potential

**DOI:** 10.3389/fpls.2014.00304

**Published:** 2014-06-25

**Authors:** Roland E. Schwarzenbacher, Estrella Luna, Jurriaan Ton

**Affiliations:** Animal and Plant Sciences Department, The University of SheffieldSheffield, UK

**Keywords:** ß-aminobutyric acid (BABA), plant defense priming, plant-pathogen interactions, aspartyl-tRNA synthetase, plant immunity

A significant proportion of global crop production is annually lost to pests and diseases (Savary et al., 2012). While pesticides help to reduce these losses, there is growing concern about pesticide resistance and their impacts on health and environment. Integrated Pest Management (IPM) aims to reduce pesticide usage through a combination of different strategies, including resistant crop cultivars, monitoring disease status, and mechanical and biological control (Birch et al., 2011; Chandler et al., 2011). A potentially novel IPM tool is plant priming agents: stimuli that sensitize the plant's immune system for augmented activation against future pathogen/herbivore attacks. Because priming leads to augmented activation of multi-genic defense mechanisms (Ton et al., 2006; Ahmad et al., 2010), the resulting resistance has the potential to be more durable than protection by single resistance (R-) genes. Despite this advantage, priming agents are not widely used in agriculture, partly because they often do not provide the same level of protection as conventional pesticides and R-genes (Walters et al., 2013). However, the advancement of IPM has spurred increased interest in exploiting priming agents in sustainable crop protection schemes.

Arguably the most effective priming agent is β-aminobutyric acid (BABA). This non-protein amino acid primes defense reactions that are controlled by salicylic acid (SA)-dependent and -independent signaling pathways (Zimmerli et al., 2000; Ton et al., 2005), conferring protection in different plant species against an exceptionally broad spectrum of stresses, including microbial pathogens, herbivores, and abiotic stresses (Jakab et al., 2001; Cohen, 2002). Unfortunately, BABA also has an undesirable side effect: it reduces plant growth (Wu et al., 2010). While this growth penalty is outweighed by its protective effects in environments with high disease pressure, it can be quite severe at higher doses under disease-free conditions (Van Hulten et al., 2006).

Until now, understanding of the molecular mechanisms underpinning the trade-off between BABA-IR and BABA-induced growth repression was limited by insufficient knowledge of how this chemical is perceived in plants. A recent study, however, has provided new insight in this matter (Luna et al., 2014). A screen for Arabidopsis mutants in BABA-IR against the biotrophic oomycete *Hyaloperonospora arabidopsidis* led to the identification of the *Impaired in BABA-induced Immunity 1* (*IBI1*) gene, encoding an aspartyl-tRNA synthetase (AspRS). Unlike previously identified genes controlling either SA-dependent, or SA-independent BABA-IR (Ton et al., 2005), the *ibi1-1* mutation was found to block both priming responses to BABA, indicating unilateral control of BABA-induced resistance by IBI1.

The stereochemical similarity between the amino acid substrate of IBI1 (L-aspartate; L-asp) and the active enantiomer of BABA (R-BABA) suggested that IBI1 might function as the BABA receptor. This hypothesis was supported by several indirect lines of evidence; apart from loss of BABA-IR by independent mutations in *IBI1*, computational models of BABA-binding to AspRS enzymes indicated high-affinity binding of R-BABA in a similar molecular orientation as L-asp, while treatment with active R-BABA caused cellular L-asp accumulation. Further evidence for receptor functionality by IBI1 came from the *in planta* demonstration that R-BABA physically binds to IBI1. It was concluded that R-BABA binds the L-asp-binding domain of IBI1, thereby disrupting canonical AspRS activity and priming the protein for non-canonical defense activity. This model also predicts that R-BABA increases uncharged tRNA^asp^ accumulation. Across eukaryotes, uncharged tRNA serves as a conserved signal for metabolic imbalance by activating the protein kinase GCN2 (Dong et al., 2000; Dever and Hinnebusch, 2005), which in turn inhibits translational activity via phosphorylation of eukaryotic translation initiation factor eIF2α (Wek et al., 2006; Li et al., 2013). Evidence that this stress pathway is activated by BABA came from the demonstration that stress-inducing concentrations of BABA activate GCN2-dependent eIF2α phosphorylation. Moreover, the *gcn2-1* mutant of Arabidopsis was strongly reduced in BABA-induced growth inhibition, while it remained unaffected in BABA-induced resistance. This latter finding not only confirmed the critical role of GCN2 in BABA-induced stress, it also demonstrated that BABA-IR and BABA-induced stress are controlled by separate pathways.

An important question from the work by Luna et al. (2014) is why plants have evolved a specific receptor protein to a xenobiotic chemical? Does BABA mimic a natural ligand, or does it induce a physiological state that is indicative of pathogen attack? An important clue came from the finding that *IBI1* transcription is increased following pathogen attack, while transgenic overexpression of *IBI1* enhances basal disease resistance through priming of inducible defenses. Hence, IBI1 can contribute to basal resistance in the absence of BABA. One of the emerging scenarios is that IBI1 contributes to basal resistance as a sensor of cellular L-aspartate (Figure 1). A sudden decline in cellular aspartate concentrations could indicate parasitization by a biotrophic pathogen and would reduce canonical AspRS activity by IBI1. It was suggested that this deprivation of canonical AspRS activity primes the alternative defense function of IBI1. This situation is mimicked by R-BABA, which blocks L-asp-IBI1 binding, tricking the protein into sensing low L-aspartate levels (Figure 1). However, it still takes a secondary stress signal after pathogen attack to fully activate the defense modus of IBI1, resulting in enhanced *IBI1* transcription, subcellular translocation of IBI1 from the ER to the cytoplasm, and augmented defense induction. Since BABA has been reported to prime PAMP-triggered immunity (PTI; Singh et al., 2012), the logical conclusion is that this secondary signal are PAMPs. Hence, IBI1 primes PTI by means of “depleted-self recognition.” This mechanism provides plants with an improved capability to recognize biotrophic pathogens, thereby counteracting their specialist ability to suppress “nonself recognition” of PAMPs (Dodds and Rathjen, 2010).

**Figure 1:**
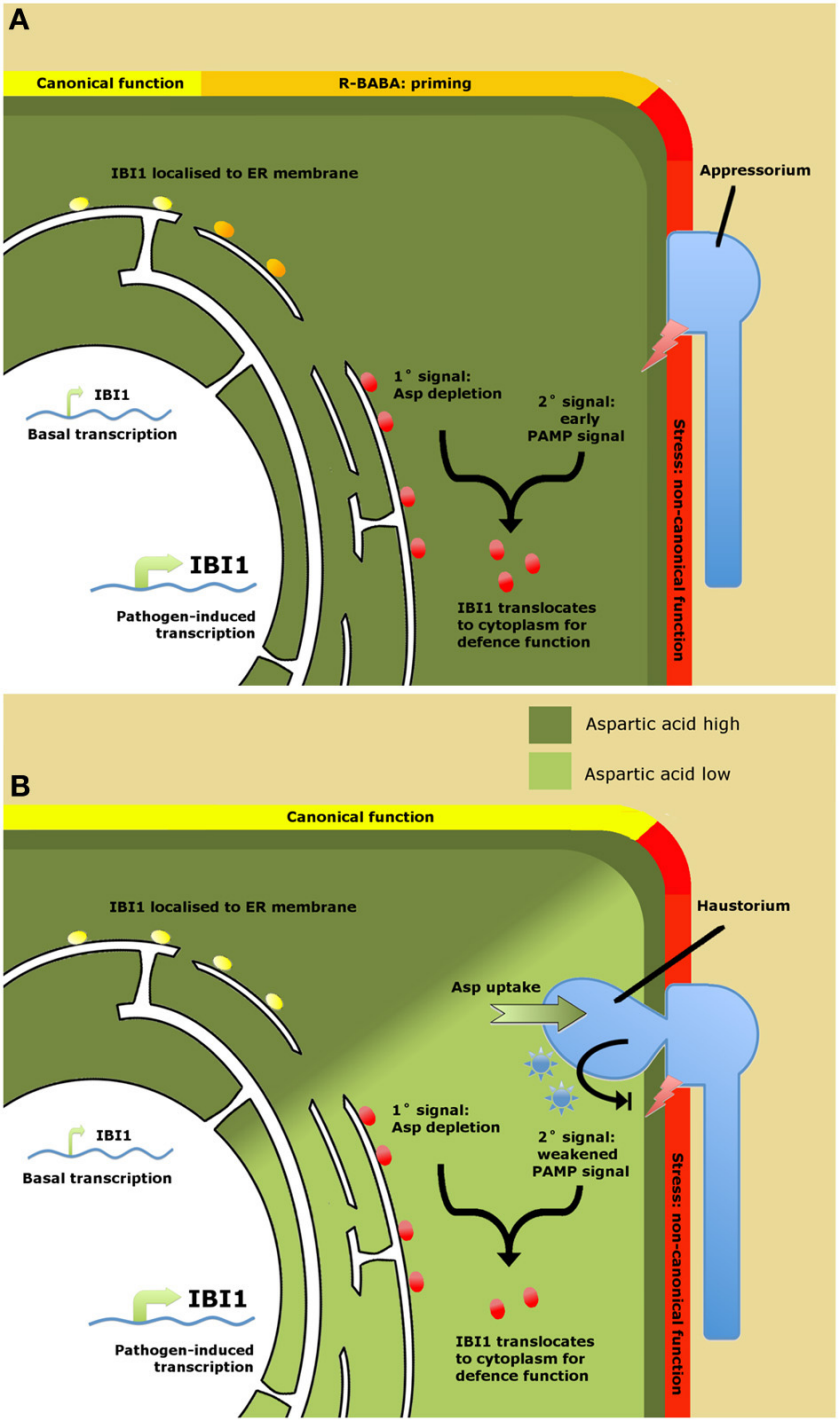
**Model of IBI1 as a regulator of BABA-induced resistance (A) and as a “depleted-self sensor” in basal resistance (B). (A)** Role of IBI1in BABA-induced resistance (BABA-IR). Binding of R-BABA to ER-localized IBI1 protein (yellow circles) deprives the protein of canonical aspartyl-tRNA synthetase (AspRS) activity, which “primes” the protein's non-canonical defense activity against pathogen attack (orange circles). Detection of pathogen-associated molecular patterns (PAMPs) during the early stages of pathogen infection boosts *IBI1* gene transcription and triggers translocation of IBI1 to the cytosol, where it activates defense activity through interaction with immune-regulatory proteins (red circles). **(B)** Role of IBI1 in basal resistance. Successful pathogen infection leads to suppression of PAMP recognition through the action of pathogen-derived effectors (blue asterisks). Ongoing parasitization and amino acid uptake by the pathogen lowers cellular L-asp levels, depriving IBI1 from its canonical AspRS activity. This, together with effector-weakened PAMP perception, boosts IBI1 transcription and elicits translocation of the protein from the ER to the cytosol, where it activates broad-spectrum defenses (red circles). Hence, IBI1 acts as a “depleted-self” sensor to counteract effector-triggered suppression of “nonself recognition.”

Luna et al. (2014) also revealed a caveat concerning agricultural exploitation of BABA: because the chemical blocks the conserved aspartate-binding domain of AspRS enzymes, it might also impact human AspRS activity. However, future research might provide opportunities to engineer constitutively primed crop varieties without relying on BABA application. It was already shown that transgenic over-expression of *IBI1* boosts basal resistance without causing phytotoxicity. This level of BABA-independent disease protection might be improved further by engineering constitutively primed IBI1 protein that is of similar configuration as BABA-bound wild-type IBI1. Such approach would not block native AspRS activity and therefore not activate the GCN2-dependent stress pathway. In summary, the study has unveiled a novel concept in plant immune regulation and given new direction toward the exploitation of defense priming in crop protection.

## Conflict of interest statement

The authors declare that the research was conducted in the absence of any commercial or financial relationships that could be construed as a potential conflict of interest.
